# Extramedullary T-lymphoblastic blast crisis in chronic myelogenous leukemia: A case report of successful diagnosis and treatment

**DOI:** 10.3892/etm.2015.2173

**Published:** 2015-01-09

**Authors:** DONG-FENG ZENG, CHENG CHANG, JIE-PING LI, PEI-YAN KONG, XI ZHANG, LEI GAO

**Affiliations:** Department of Hematology, Xinqiao Hospital, The Third Military Medical University, Chongqing 400037, P.R. China

**Keywords:** extramedullary blast crisis, chronic myelogenous leukemia, stem cell transplantation

## Abstract

Extramedullary T-lymphoblastic blast crisis of chronic myelogenous leukemia (CML) is uncommon and the prognosis is poor. It was usually misdiagnosed as the co-existence of T-lymphoblastic lymphoma (T-LBL) and CML. In the present study, we report a patient with CML, who developed extramedullary T-lymphoblastic blast crisis and was successfully treated with human leukocyte antigen (HLA)-mismatched stem cell transplantation. The patient was a 44-year-old man who presented with lymphadenectasis and leucocytosis prior to diagnosis. The bone marrow smear, biopsy and fluorescence *in situ* hybridization (FISH) of Breakpoint Cluster Region/ Abelson murine leukaemia (BCR/ABL) supported the diagnosis of CML in the chronic phase, while the immunohistochemistry of lymph nodes supported the diagnosis of T-LBL. The FISH test for BCR/ABL in lymph node blast cells was performed and the result was positive; therefore, the patient was diagnosed with extramedullary T-lymphoblastic blast crisis of CML. After several courses of combined chemotherapy, the patient was treated with HLA-mismatched stem cell transplantation and obtained continuous remission for 51 months until the present (September 2013).

## Introduction

Chronic myelogenous leukemia (CML) is the most common myeloproliferative disease, and the majority of cases of CML have a (9;22) cytogenetic disorder, resulting in Breakpoint Cluster Region/ Abelson murine leukaemia (BCR/ABL) fusion which activates tyrosine kinase and leads to the uncontrolled proliferation of myeloid cells (1–3). The clinical course of CML is characterized by three phases: the chronic phase, the accelerated phase and blast crisis phase. The blast crisis phase is accompanied by an increasing percentage of blast cells in the peripheral blood and bone marrow, while certain patients with CML develop extramedullary blast crisis (EBC) caused by the extramedullary infiltration of blast cells (4–6). CML-EBC is rare with a poor prognosis and is may be difficult to distinguish from the co-existence of two hematological neoplasms.

Few cases concerning extramedullary T-lymphoblastic blast crisis of CML have been reported in the literature, and the outcome of EBC, even with allogeneic stem cell transplantation, is poor (5–11). In this study, we report the case of a patient with extramedullary T-lymphoblastic blast crisis of CML treated with human leukocyte antigen (HLA)-mismatched stem cell transplantation who remained in complete remission for 51 months. The study was approved by the Ethics Committee of The Third Military Medical University (Chongqing, China).

## Case report

**Figure 1:**
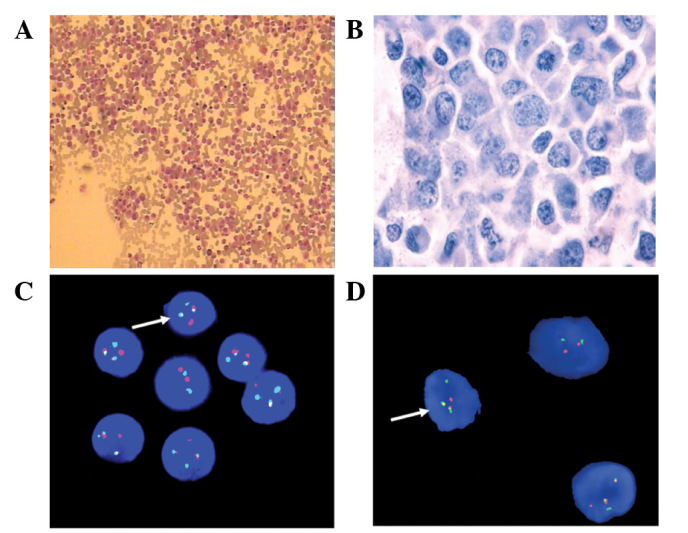
(A) Bone marrow smear showed a myeloproliferative disorder with hyperactivity in the medullary system, while the myeloblast and promyelocyte cells were <5%. (B) Bone marrow biopsy revealed a myeloproliferative disorder with hyperactivity in the medullary system. (C) FISH analysis of bone marrow cells was positive for the BCR/ABL infusion gene. (D) FISH testing of lymph node cells was also positive for the BCR/ABL infusion gene. FISH, fluorescence *in situ* hybridization. The arrows show two red dots, two yellow dots and two green dots, positive for the BCR/ABL infusion gene.

The patient was a 44-year-old male who presented in March 2009 with palpitations, dyspnea and lymphadenectasis in the neck. The patient was treated with antibiotics for one week, during which time the symptoms were not alleviated. A subsequent ultrasonic examination showed several low echo-level masses in the bilateral axillary, submaxillary regions, neck and supraclavicular fossa. The largest mass was 3.0×2.2 cm in size and the blood supply was abundant. An enlarged spleen and liver were also detected. A complete blood count (CBC) test obtained the following results: hemoglobin 10.2 g/dl, leukocyte count 169.87×10^9^ cells/l, (neutrophils, 84.93×10^9^ cells/l; lymphocytes, 1.70×10^9^ cells/l; monocytes, 5.07×10^9^ cells/l; eosinophils, 0.84×10^9^ cells/l; basophils, 2.55×10^9^ cells/l) and platelet count 129×10^9^ cells/l. Bone marrow analysis revealed a myeloproliferative disorder with hyperactivity in the medullary system (Fig. 1A and B). The fluorescence *in situ* hybridization (FISH) test for BCR/ABL was positive (Fig. 1C). The patient was diagnosed with CML in the chronic phase, and hydroxyurea was then administered from March 5, 2009. Informed consent was obtained from the patient prior to this study.

**Figure 2:**
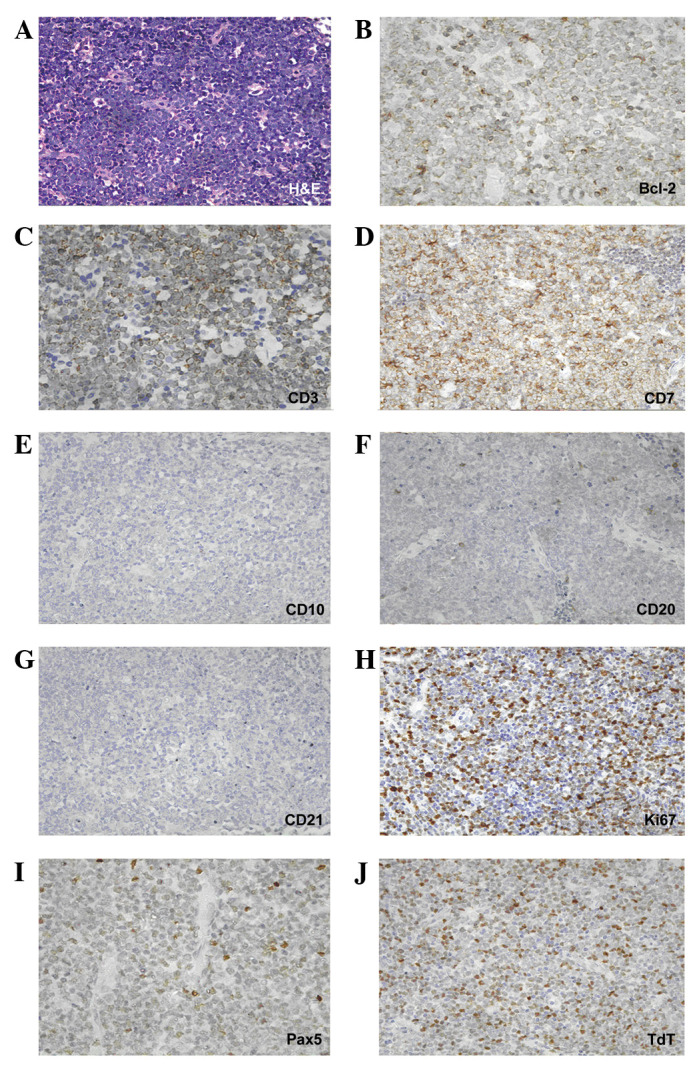
(A) High magnification showed that lymph node cells were replaced by blast cells (hematoxylin and eosin; magnification, ×400). Immunostaining in blasts was positive for (B) Bcl-2 antigen (magnification, ×630), (C) CD3 antigen (magnification, ×630) and (D) CD7 antigen (magnification, ×630), (E) negative for CD10 antigen (magnification, ×400), (F) low level positive for CD20 antigen (magnification, ×400), (G) negative for CD21 antigen (magnification, ×400), (H) positive for Ki-67 antigen (magnification, ×400) with a labeling index of 70%, (I) positive for PAX5 antigen (magnification, ×630) and (J) positive for terminal deoxynucleotidyl transferase (TdT; magnification, ×400).

When the ultrasonic examination was performed, the patient underwent a left cervical lymph node biopsy. Immunohistochemical staining showed that the lymphoblastic cells expressed CD7, CD3, PAX5, Bcl-2, terminal deoxynucleotidyl transferase (TdT) and, to a lesser extent, CD20. By contrast, staining for CD10 and CD21 was negative. The Ki-67/MIB-1 labeling index was 70% (Fig. 2). A preliminary diagnosis of a precursor T-lymphoblastic lymphoma (T-LBL) was made. A week later, the FISH test for BCR/ABL was positive in lymph node section (Fig. 1D), and the final diagnosis of EBC of CML was made.

The initial chemotherapy regimen CHOP was administered from March 21, 2009 and, after the first course of chemotherapy, the patient’s lymphadenectasis disappeared. Two further courses of the chemotherapy regimen followed between April and May 2009. Hematopoietic stem cell transplantation (HSCT) from a matched relative donor had been considered, but no donor was identified. While the patient’s sister was revealed to be HLA-mismatched donor, only HLA-DQ was mismatched. The allo-HSCT was administered from May 27, 2009 with the conditioning regimen: Bu/CY/Ara-c/CCNU (BU, busulfan; CY, cyclophosphamide; Ara-c, aracytidine; CCNU, lomustine). On June 3, 2009 peripheral blood stem cells were collected from the donor and infused into the patient. The following day, bone marrow was collected from the donor and administered to the patient by infusion. During the transplantation period, anti-graft-versus-host disease (anti-GVHD) medications, including CsA, MTX, mycophenolate mofetil and anti-thymocyte globulin (ATG) were administered to the patient. Four weeks later, hematopoietic reconstitution was definite. Accompanied by the chemotherapy, the patient received lumbar puncture and intrathecal chemotherapy eight times, and the cerebrospinal fluid was continuously normal.

Two months after transplantation, the patient received a repeated ultrasonic examination and the results showed that lymphadenectasis had disappeared and the sizes of spleen and liver were normal. FISH detection demonstrated changes in the patient’s sex chromosomes from XY to XX and the test for BCR/ABL was negative. Until now (September 2013)the patient obtained continuous remission for 51 months and recent ultrasonic examination, FISH detection, bone marrow smear and biopsy results were normal.

## Discussion

Blast crisis phase is the terminal stage of CML and it constitutes a different form of CML. EBC is a special form of blast crisis and has been observed in <10% of patients with CML (12–14). EBC commonly occurs in bone, skin, lymph nodes and certain other soft tissues, even though the bone marrow of CML patients is still in the chronic stage (7,11). The majority of Blast Crisis (BC) cells of CML are of myeloid lineage; therefore, when the biopsy of the lymph node or other extramedullary tissue reveals the evidence of myeloid origin and the BCR/ABL fusion gene is positive, the diagnosis is straightforward. When the EBC cells are of lymphoid lineage and the lymphadenopathy is the only symptom prior to final diagnosis, it may be misdiagnosed as lymphoma. In the current case, the biopsy of the lymph node showed that the lymphoblastic cells expressed CD7, CD3, PAX5, Bcl-2 and TdT, whereas the tests for CD10 and CD21 were negative. The Ki-67/MIB-1 labeling index was 70%. Initially, we made a misdiagnosis of precursor T-LBL accompanied by CML. Subsequently, we obtained a positive result for BCR/ABL in the lymph node by FISH test and the final diagnosis of EBC of CML was made. Ichinohasama *et al* reported two cases of Ph-negative non-Hodgkin’s lymphoma (NHL) occurring in CML and reviewed the literature concerning Ph^+^ and Ph^−^ lymphoma (15); the FISH of BCR/ABL was considered to be the essential test for blast crisis of CML while the PCR of BCR/ABL was not. The majority of cases of so-called ‘Ph^+^ lymphoma’ occurred in patients without a diagnosis of CML or acute lymphocytic leukemia (ALL) and it was difficult to state whether the NHL carried the BCR/ABL gene translocation without FISH.

The majority of cases of EBC occur several months or years after the diagnosis of CML and the relevant therapy, including hydroxycarbamide and imatinib, has been regarded as the cause of EBC (5). Kim *et al* reported two cases who received imatinib therapy after being diagnosed with CML in the chronic phase (5). After 3–5 months, the two patients presented with lymphadenectasis and the enlarged lymph nodes were subsequently excised and revealed T-cell-type acute lymphatic leukemia (T-ALL). However, in the present study the EBC was diagnosed together with CML in chronic phase, so the exact pathogenesis of EBC awaits further clarification.

The prognosis of EBC is poor, as the majority of patients succumb within 6–8 months of the final diagnosis (7–10). Hydroxycarbamide is effective for the treatment of leucocytosis and lymphadenectasis, but is capable of changing the pathogenesis of CML. Imatinib is able to target tyrosine kinase and decrease the synthesis of BCR/ABL fusion gene, Naito *et al* reported their experience of treating a patient with CML accompanied by EBC with imatinib and obtained favorable results (16). Another study has shown that CML patients in the chronic phase without EBC may eventually acquire EBC during imatinib therapy (5). Based on the evidence above, the effects of imatinib on EBC remain uncertain. In the present case, hydroxycarbamide and combined sequential chemotherapy were administered first and followed by a successful HLA-mismatched HSCT. The patient obtained genetic remission from CML and no lymphadenectasis was observed following hematopoietic reconstitution. The continuous remission status was maintained for 51 months till now (September 2013).

In conclusion, extramedullary T-lymphoblastic blast crisis of CML as a first diagnosis is rare and may be misdiagnosed as lymphoma without BCR/ABL detection by FISH. Despite the view that allografts may be the cause of an extramedullary relapse or blast crisis for CML (10), allo-HSCT therapy may be an effective therapy for EBC of CML.
